# Subtyping of Breast Cancer by Immunohistochemistry to Investigate a Relationship between Subtype and Short and Long Term Survival: A Collaborative Analysis of Data for 10,159 Cases from 12 Studies

**DOI:** 10.1371/journal.pmed.1000279

**Published:** 2010-05-25

**Authors:** Fiona M. Blows, Kristy E. Driver, Marjanka K. Schmidt, Annegien Broeks, Flora E. van Leeuwen, Jelle Wesseling, Maggie C. Cheang, Karen Gelmon, Torsten O. Nielsen, Carl Blomqvist, Päivi Heikkilä, Tuomas Heikkinen, Heli Nevanlinna, Lars A. Akslen, Louis R. Bégin, William D. Foulkes, Fergus J. Couch, Xianshu Wang, Vicky Cafourek, Janet E. Olson, Laura Baglietto, Graham G. Giles, Gianluca Severi, Catriona A. McLean, Melissa C. Southey, Emad Rakha, Andrew R. Green, Ian O. Ellis, Mark E. Sherman, Jolanta Lissowska, William F. Anderson, Angela Cox, Simon S. Cross, Malcolm W. R. Reed, Elena Provenzano, Sarah-Jane Dawson, Alison M. Dunning, Manjeet Humphreys, Douglas F. Easton, Montserrat García-Closas, Carlos Caldas, Paul D. Pharoah, David Huntsman

**Affiliations:** 1 Department of Oncology, University of Cambridge, United Kingdom; 2 Netherlands Cancer Institute, Amsterdam, The Netherlands; 3 Department of Pathology and Laboratory Medicine, The University of British Columbia, Vancouver, Canada; 4 Department of Oncology, Helsinki University Central Hospital, Helsinki, Finland; 5 Department of Pathology, Helsinki University Central Hospital, Helsinki, Finland; 6 Department of Obstetrics and Gynecology, Helsinki University Central Hospital, Helsinki, Finland; 7 The Gade Institute, Section for Pathology, University of Bergen, Haukeland University Hospital, Bergen, Norway; 8 Department of Pathology, McGill University and Hôpital du Sacré-Coeur de Montréal, Montreal, Quebec, Canada; 9 Program in Cancer Genetics, Departments of Oncology and Human Genetics, McGill University, Montreal, Quebec, Canada; 10 Department of Laboratory Medicine and Pathology, Mayo Clinic, Rochester, Minnesota, United States of America; 11 Department of Health Sciences Research, Mayo Clinic, Rochester, Minnesota, United States of America; 12 Cancer Epidemiology Centre, The Cancer Council Victoria, Melbourne, Australia; 13 The Alfred Hospital, Melbourne, Australia; 14 Genetic Epidemiology Laboratory, Department of Pathology, The University of Melbourne, Australia; 15 Departments of Histopathology and Surgery, The Breast Unit, Nottingham City Hospital NHS Trust and University of Nottingham, Nottingham, United Kingdom; 16 Division of Cancer Epidemiology and Genetics, National Cancer Institute, Rockville, Maryland, United States of America; 17 M. Sklodowska-Curie Memorial Cancer Center & Institute of Oncology, Warsaw, Poland; 18 Institute for Cancer Studies, University of Sheffield School of Medicine, Sheffield, United Kingdom; 19 Academic Unit of Pathology, University of Sheffield School of Medicine, Sheffield, United Kingdom; 20 Academic Unit of Surgical Oncology, University of Sheffield School of Medicine, Sheffield, United Kingdom; 21 Department of Public Health and Primary Care, University of Cambridge, Cambridge, United Kingdom; National Institutes of Health, United States of America

## Abstract

**Background:**

Immunohistochemical markers are often used to classify breast cancer into subtypes that are biologically distinct and behave differently. The aim of this study was to estimate mortality for patients with the major subtypes of breast cancer as classified using five immunohistochemical markers, to investigate patterns of mortality over time, and to test for heterogeneity by subtype.

**Methods and Findings:**

We pooled data from more than 10,000 cases of invasive breast cancer from 12 studies that had collected information on hormone receptor status, human epidermal growth factor receptor-2 (HER2) status, and at least one basal marker (cytokeratin [CK]5/6 or epidermal growth factor receptor [EGFR]) together with survival time data. Tumours were classified as luminal and nonluminal tumours according to hormone receptor expression. These two groups were further subdivided according to expression of HER2, and finally, the luminal and nonluminal HER2-negative tumours were categorised according to expression of basal markers. Changes in mortality rates over time differed by subtype. In women with luminal HER2-negative subtypes, mortality rates were constant over time, whereas mortality rates associated with the luminal HER2-positive and nonluminal subtypes tended to peak within 5 y of diagnosis and then decline over time. In the first 5 y after diagnosis the nonluminal tumours were associated with a poorer prognosis, but over longer follow-up times the prognosis was poorer in the luminal subtypes, with the worst prognosis at 15 y being in the luminal HER2-positive tumours. Basal marker expression distinguished the HER2-negative luminal and nonluminal tumours into different subtypes. These patterns were independent of any systemic adjuvant therapy.

**Conclusions:**

The six subtypes of breast cancer defined by expression of five markers show distinct behaviours with important differences in short term and long term prognosis. Application of these markers in the clinical setting could have the potential to improve the targeting of adjuvant chemotherapy to those most likely to benefit. The different patterns of mortality over time also suggest important biological differences between the subtypes that may result in differences in response to specific therapies, and that stratification of breast cancers by clinically relevant subtypes in clinical trials is urgently required.

*Please see later in the article for the Editors' Summary*

## Abstract 2

Paul Pharoah and colleagues evaluate the prognostic significance of immunohistochemical subtype classification in more than 10,000 breast cancer cases with early disease, and examine the influence of a patient's survival time on the prediction of future survival.

## Editors' Summary

**Background:**

Each year, more than one million women discover they have breast cancer. Breast cancer begins when cells in the breast's milk-producing glands or in the tubes (ducts) that take milk to the nipples acquire genetic changes that allow them to divide uncontrollably and to move around the body (metastasize). The uncontrolled cell division leads to the formation of a lump that can be detected by mammography (a breast X-ray) or by manual breast examination. Breast cancer is treated by surgical removal of the lump or, if the cancer has started to spread, by removal of the whole breast (mastectomy). Surgery is usually followed by radiotherapy or chemotherapy. These “adjuvant” therapies are designed to kill any remaining cancer cells but can make women very ill. Generally speaking, the outlook (prognosis) for women with breast cancer is good. In the United States, for example, nearly 90% of affected women are still alive five years after their diagnosis.

**Why Was This Study Done?:**

Because there are several types of cells in the milk ducts and glands, there are several subtypes of breast cancer. Luminal tumors, for example, begin in the cells that line the ducts and glands and usually grow slowly; basal-type tumors arise in deeper layers of the ducts and glands and tend to grow quickly. Clinicians need to distinguish between different breast cancer subtypes so that they can give women a realistic prognosis and can give adjuvant treatments to those women who are most likely to benefit. One way to distinguish between different subtypes is to stain breast cancer samples using antibodies (immune system proteins) that recognize particular proteins (antigens). This “immunohistochemical” approach can identify several breast cancer subtypes but its prognostic value and the best way to classify breast tumors remains unclear. In this study, the researchers investigate the survival over time of women with six major subtypes of breast cancer classified using five immunohistochemical markers: the estrogen receptor and the progesterone receptor (two hormone receptors expressed by luminal cells), the human epidermal growth factors receptor-2 (HER2, a protein marker used to select specific adjuvant therapies), and CK5/6 and EGFR (proteins expressed by basal cells).

**What Did the Researchers Do and Find?:**

The researchers pooled data on survival time and on the expression of the five immunohistochemical markers from more than 10,000 cases of breast cancer from 12 studies. They then divided the tumors into six subtypes on the basis of their marker expression: luminal (hormone receptor-positive), HER2-positive tumors; luminal, HER2-negative, basal marker-positive tumors; luminal, HER2-negative, basal marker-negative tumors; nonluminal (hormone receptor-negative), HER2-positive tumors; nonluminal, HER2-negative, basal marker-positive tumors; and nonluminal, HER2-negative, basal marker-negative tumors. In the first five years after diagnosis, women with nonluminal tumor subtypes had the worst prognosis but at 15 years after diagnosis, women with luminal HER2-positive tumors had the worst prognosis. Furthermore, death rates (the percentage of affected women dying each year) differed by subtype over time. Thus, women with the two luminal HER2-negative subtypes were as likely to die soon after diagnosis as at later times whereas the death rates associated with nonluminal subtypes peaked within five years of diagnosis and then declined.

**What Do These Findings Mean?:**

These and other findings indicate that the six subtypes of breast cancer defined by the expression of five immunohistochemical markers have distinct biological characteristics that are associated with important differences in short-term and long-term outcomes. Because different laboratories measured the immunohistochemical markers using different methods, it is possible that some of the tumors included in this study were misclassified. However, the finding of clear differences in the behavior of the immunochemically classified subtypes suggests that the use of the five markers for tumor classification might be robust enough for routine clinical practice. The application of these markers in the clinical setting, suggest the researchers, could improve the targeting of adjuvant therapies to those women most likely to benefit. Furthermore, note the researchers, these findings strongly suggest that subtype-specific responses should be evaluated in future clinical trials of treatments for breast cancer.

**Additional Information:**

Please access these Web sites via the online version of this summary at http://dx.doi.org/10.1371/journal.pmed.1000279.

- This study is further discussed in a *PLoS Medicine* Perspective by Stefan Ambs
- The US National Cancer Institute provides detailed information for patients and health professionals on all aspects of breast cancer (in English and Spanish)
- The American Cancer Society has a detailed guide to breast cancer, which includes information on the immunochemical classification of breast cancer subtypes
- The UK charities MacMillan Cancer Support and Cancer Research UK also provide detailed information about breast cancer
- The MedlinePlus Encyclopedia provides information for patients about breast cancer; Medline Plus provides links to many other breast cancer resources (in English and Spanish)

## Introduction

Breast cancer is a heterogeneous disease that can be classified using a variety of clinical and pathological features. Classification may help in prognostication and targeting of treatment to those most likely to benefit. Currently, estrogen receptor (ER) status and human epidermal growth factor receptor-2 (HER2) status are routinely used as predictive markers to select specific adjuvant therapies. Prognostic markers may also be used to target adjuvant chemotherapy to those at highest risk of poor outcome—for example, the risk prediction tool Adjuvant!Online (www.adjuvant.org) uses prognostic markers to predict the likely absolute benefit of postoperative hormonal and/or chemotherapy and is widely used by oncologists to identify patients most likely to benefit from adjuvant treatment.

Perou et al. identified four breast cancer subtypes on the basis of gene-expression profiling of 39 invasive breast tumours and three normal breast specimens [1]. There was one ER-positive (ER+/luminal-like) and three ER-negative subtypes (basal-like, ERBB2+, and normal-like). In addition to expressing the ER receptor, luminal-like tumours expressed other genes that were characteristic of luminal or glandular epithelial cells of origin. The basal-like tumours expressed basal or myoepithelial markers, and none of the basal tumours expressed ER. Similar to the basal-like tumours, overexpression of the *ERBB2* oncogene was associated with low ER. The normal-like subgroup was typified by high gene expression for basal and low expression for luminal breast epithelium. A subsequent gene expression analysis by Sorlie et al. of patterns in 78 breast cancers, three fibroadenomas, and four normal breast tissues suggested that the luminal-like subtype could be further separated into two subgroups: luminal A and luminal B [2]. The molecular subtypes were reflected in differences in prognosis. Overall and relapse-free survivals were most favourable for luminal A tumours and least favourable for ERBB2+ and basal-like breast cancers. The investigators also suggested that there may be a third luminal subgroup, the luminal C tumours, but this has not been supported by the subsequent analysis of an expanded dataset [3].

The classification of breast cancers into subgroups on the basis of gene expression patterns in tumour tissue is often regarded as the gold standard, but widespread use of gene-expression profiling in either the clinical or the research setting remains limited. Lack of widespread use of expression profiles is primarily due to the expense and technical difficulty encountered when carrying out high-throughput gene-expression profiling using paraffin-embedded material. Moreover, the currently defined subtypes based on expression profiling were determined through the study of relatively small numbers of tumours and these subgroups may not be definitive. Consequently there is interest in using immunohistochemical (IHC) markers to classify tumours into subtypes that are surrogates for those based on gene-expression profiling [4].

Many investigators have used IHC to classify tumours but have used different naming conventions. Generally a hierarchical classification is used, with luminal and nonluminal tumours defined as those tumours that express either ER or progesterone receptor (PR) and those that do not. The luminal and nonluminal groups can then be further subdivided according to HER2-expression status to generate four subtypes, and these four subtypes can each be categorised according to whether or not they express a basal marker yielding a total of eight subtypes. The mapping of these eight IHC subtypes onto the five subtypes based on gene expression is not exact.

Luminal A tumours as defined by gene expression have, in general, higher expression of ER-related genes and lower expression of proliferative genes than luminal B tumours [5]. However, there are no established IHC markers for subdividing the luminal subtypes into the same categories. Recently, it has been suggested that the luminal B subtype is equivalent to those that express either HER2 or the proliferation marker KI67 [6]. The nonluminal tumours are ER negative and PR negative and are generally subdivided into three groups. The nonluminal, HER2-positive tumours are the equivalent of the ERBB2-overexpressing tumours. Tumours that do not express ER, PR, or HER2—the triple negative phenotype (TNP) tumours—are often regarded as equivalent to the basal subtype as they can be easily identified with IHC markers that are currently used in routine clinical use. However, not all TNP tumours express basal cytokeratins (CKs), and within the TNP subtype, expression of basal markers may reflect important clinical differences. Expression of either CK5/6 or epidermal growth factor receptor (EGFR) has been shown to accurately identify basal-like tumours classified using gene expression [7],[8], and several published studies have used these markers to subclassify the TNP tumours into a core basal subgroup (CBP), which is equivalent to the basal-like from expression profiling and the five negative phenotype (5NP: ER−, PR−, HER2−, CK5/6−, and EGFR−). Although this hierarchical classification is commonly used, questions remain as to whether these groups are biologically distinct and clinically relevant. For example, it has been suggested that basal markers can be used to classify the basal tumours independent of other markers [9]. Cheang et al. reported a significantly poorer survival in CBP tumours compared to the 5NP tumours [10], an observation that supports the notion that the two are biologically distinct types of the TNP tumours. This finding was not confirmed by a smaller study with limited power to detect small differences [11]. A third study reported that the prognostic significance of CBP tumours was similar to that of the TNP tumours [12]. However, they did not explicitly compare the CBP and 5NP subtypes.

Previously published studies have either compared the five subtypes by using the luminal HER2-negative tumours as a reference category to compare with the other four subtypes [8],[10],[12],[13], or they have compared the subtypes by restricting the analysis to either luminal or nonluminal tumours [6],[11]. Unanswered questions include whether the behaviour of luminal HER2-positive tumours and the nonluminal HER2-positive tumours are different, whether the behaviour of luminal basal-positive tumours is different from that of the nonluminal basal-positive tumours, and whether basal marker status is important in the luminal, HER2-negative tumours.

The association between ER status and mortality is known to be time dependent, with hazard ratios for ER-positive versus ER-negative tumours being lower than one in the first years after diagnosis and becoming higher than one after 7–10 y. Mortality in women with ER-positive tumours remains fairly constant over time, whereas the mortality in women with ER-negative tumours is initially higher than that in women with ER-positive disease and then falls to a lower rate after 7–10 y [14]–[16]. In addition, Tischkowitz and colleagues reported that the prognostic effects of both TNP and CBP tumours compared to luminal tumours tended to diminish over time, whereas the effect of CK5 and other basal markers, when considered alone, might increase with time [12]. Another study reported that the effects of the CBP were attenuated over time [13]. Inspection of the Kaplan-Meier survival curves published by Cheang et al. also suggest that the prognostic effects of the CBP and 5NP subtypes are time dependent [10].

All the major subtypes apart from the luminal A tumours are relatively infrequent, and only very large studies with prolonged follow-up have the power to study meaningful differences in prognosis. The aim of this study was to pool individual data from multiple breast cancer case series, in order to definitively establish the relative survival of the major subtypes of breast cancer as classified using five IHC markers, and to characterise their prognostic effects over time.

## Materials and Methods

### Ethics Statement

All studies were approved by the relevant research ethics committee or institutional review board. Participants in Amsterdam Breast Cancer Study (ABCS), Helsinki Breast Cancer Study (HEBCS), Jewish General Hospital (JGH), Mayo Clinic Breast Cancer Study (MCBCS), Melbourne Collaborative Cohort Study (MCCS), Polish Breast Cancer Study (PBCS), Sheffield Breast Cancer Study (SBCS), and Study of Epidemiology and Risk factors in Cancer Heredity (SEARCH) provided informed written consent. Samples for British Columbia Cancer Agency (BCCA), Nottingham Breast Cancer Case Series (NOBCS), University of British Columbia (UBC), and Vancouver General Hospital (VGH) were from legacy archival material and individual consent was not obtained. All data were anonymised before being sent to the coordinating centre for analysis.

### Study Populations

The international breast cancer association consortium (BCAC) comprises a large number of studies investigating the role of common germline genetic variation in breast cancer susceptibility [17]. In addition to data on germline genotype, many BCAC studies have detailed pathological data on the breast cancer cases linked to follow-up data. All BCAC studies that had collected IHC data on ER, PR, HER2, and either EGFR or CK5/6 or both, in addition to survival time data and data on tumour grade, size, and nodal status were eligible for inclusion in this study. The investigators of the three previously published studies with equivalent data [10]–[12], were also invited to contribute their data, as were the investigators of a fourth large breast cancer case series that had taken part in a previous collaboration involving other BCAC studies [18]. All studies provided data on age at diagnosis, vital status, breast cancer-specific mortality, time between diagnosis and ascertainment, follow-up time, tumour grade (low, intermediate, and high), tumour size (<2 cm, 2–4.9 cm, ≥5 cm) and node status (positive or negative). In total, 12 studies from Europe, North America, and Australia contributed data on 10,159 cases with complete data [7],[9],[10],[12],[18]–[29]. Nine studies also provided data on whether or not the patient had been treated with adjuvant hormonal therapy or adjuvant chemotherapy. These data were available for a subset of 8,171 and 8,061 cases, respectively. The studies are described in Table 1.

**Table 1:** Description of participating studies.

Study	Country	Case Ascertainment	Case Definition	Age Range (y)	References
**ABCS**	The Netherlands	Hospital-based	All cases of operable, invasive cancer diagnosed from 1974 to 1994 in four Dutch hospitals. Familial non-BRCA1/2 cases <50 from the Clinical Genetic Centre at The Netherlands Cancer Institute	23–50	[19]
**BCCA**	Canada	Hospital-based	Women diagnosed with invasive breast cancer between 1986 to 1992 and identified through the British Columbia Cancer Agency	23–89	[7],[10]
**HEBCS**	Finland	Hospital-based	(1) Consecutive cases (883) from the Department of Oncology, Helsinki University Central Hospital 1997–1998 and 2000; (2) Consecutive cases (986) from the Department of Surgery, Helsinki University Central Hospital 2001–2004; (3) Familial breast cancer patients (536) from the Helsinki University Central Hospital, Departments of Oncology and Clinical Genetics (1995–)	22–96	[20]–[22]
**JGH**	Canada	Hospital-based	Ashkenazi Jewish women diagnosed with nonmetastatic, invasive breast cancer at Jewish General Hospital, Montreal between 1980 and 1995	26–66	[12]
**MCBCS**	USA	Hospital-based	Incident cases residing in six states (Minnesota, Wisconsin, Iowa, Illinois, North Dakota, South Dakota) seen at the Mayo Clinic in Rochester, Minnesota from 2002–2005	22–89	[23]
**MCCS**	Australia	Cohort	Incident cases diagnosed within the Melbourne Collaborative Cohort Study during the follow-up from baseline (1990–1994) to 2004 of the 24,469 participating women	30–82	[24]
**NOBCS**	UK	Hospital-based	Primary operable breast carcinoma patients presenting from 1986 to 1998 and entered into the Nottingham Tenovus Primary Breast Carcinoma Series.	26–93	[9]
**PBCS**	Poland	Population-based	Incident cases from 2000–2003 identified through a rapid identification system in participating hospitals covering ∼90% of all eligible cases; periodic check against the cancer registries in Warsaw and Łódź to assure complete identification of cases	27–75	[25]
**SBCS**	UK	Hospital-based	Women with pathologically confirmed breast cancer recruited from surgical outpatient clinics at the Royal Hallamshire Hospital, Sheffield, 1998–2002; cases are a mixture of prevalent and incident disease	29–93	[26],[27]
**SEARCH**	UK	Population-based	Two groups of cases identified through East Anglian Cancer Registry: (1) prevalent cases diagnosed age <55 y from 1991–1996 and alive when study started in 1996; (2) incident cases diagnosed age <70 y diagnosed after 1996	23–69	[18]
**UBCBCT**	Canada	Hospital-based	Women with stage I to III breast cancer who participated in four different British Columbia Cancer Agency clinical trials between 1970 and 1990 and all received chemotherapy	22–90	[28],[29]
**VGH**	Canada	Hospital-based	Women with primary breast cancer who underwent surgery at Vancouver General Hospital 1975–1995	28–91	[12]

ABCS, Amsterdam Breast Cancer Study; BCCA, British Columbia Cancer Agency; HEBCS, Helsinki Breast Cancer Study; JGH, Jewish General Hospital; MCBCS, Mayo Clinic Breast Cancer Study; MCCS, Melbourne Collaborative Cohort Study; NOBCS, Nottingham Breast Cancer Case Series; PBCS, Polish Breast Cancer Study; SBCS, Sheffield Breast Cancer Study; SEARCH, Study of Epidemiology and Risk factors in Cancer Heredity; UBCBCT, University of British Columbia Breast Cancer Trials; VGH, Vancouver General Hospital.

### Immunohistochemistry and Tumour Classification

**Figure 1:**
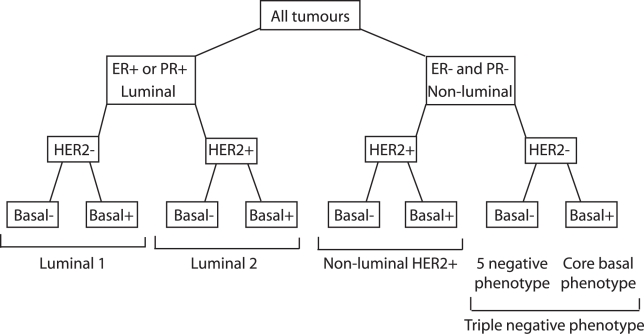
Classification of breast cancer subtypes according to IHC marker profile.

Data for these antibodies were either derived from IHC performed in a research setting or collated from patient records by the individual groups. The methods used by each study for each marker are shown in Table S1. The cases were grouped into subtypes on the basis of their protein expression profile (Figure 1). Luminal tumours were those with positive staining for ER or PR. Luminal tumours were subdivided according to HER2 status into luminal 1 (HER2-negative), which is broadly equivalent to the luminal A tumours defined by gene expression, and luminal 2 (HER2-positive) tumours. The luminal 2 tumours are a subset of the luminal B tumours because some of the tumours classified as luminal 1 would be expected to express proliferative markers and thus be misclassified luminal B tumours. The nonluminal tumours were those that were negative for both ER and PR. These were subdivided by HER2 expression status into the nonluminal HER2-positive tumours and the TNP tumours. The TNP tumours were further subdivided into the CBP tumours (either CK5/6 or EGFR positive) and the 5NP tumours (CK5/6-negative and EGFR-negative). Four studies did not provide data for EGFR, and for these studies the 5NP tumours were those that were negative for ER, PR, HER2, and CK5/6. A small number of 5NP tumours from these studies will thus be misclassified core basal tumours. The tumours classified as luminal 1 were also further subdivided according to expression of basal markers into luminal 1, basal marker negative and luminal 1, basal marker positive.

### Statistical Analysis

The association between each prognostic marker and subtype and all-cause mortality after diagnosis was investigated using Cox regression stratified by study and adjusted for age at diagnosis, grade, node status, and size of tumour. Ordinal categories of tumour grade and size were treated as continuous variables in all analyses. Age at diagnosis was treated as a categorical variable (<40, 40–49, 50–59, and ≥60 y). In several studies the cases were ascertained after diagnosis (prevalent cases), and this was allowed for in the analysis by setting “time at risk” from the date of diagnosis and “time under observation” on date of study entry. This step produces an unbiased estimate of the hazard ratio provided the proportional hazards assumption is correct [16]. Follow-up was censored on the date of death from any cause, or, if death did not occur, on date last known alive or at 15 y after diagnosis, whichever came first. The Cox proportional hazards model assumes that the hazard ratio is constant over time. This assumption is known to be violated for ER [14]–[16] and over prolonged follow-up is also likely to be violated for other predictors. We therefore carried out a conditional relative survival analysis by splitting follow-up time into five different periods—0–2, 2–4, 4–6, 6–10, and 10–15 y after diagnosis—and deriving Cox models separately for each period. The Cox proportional hazards assumption was checked for each study period by visual inspection of the standard log-log plots. A test for heterogeneity of the study-specific hazard ratios was carried out using the Mantel-Haenszel method. Kaplan-Meier cumulative survival plots were adjusted for study, age group, tumour grade, tumour size, and node status. In order to provide an overall test of association to compare survival time across all 15 y of follow-up we used multivariate Cox regression models in which the prognostic factors were treated as time-varying covariates. In these models the log hazard ratio varies as a function of the natural logarithm of follow-up time. Models with and without the covariates of interest were then compared using likelihood ratio tests. All analyses were performed in Intercooled Stata, version 10 (Stata Corp).

## Results

Eight studies provided data on ER, PR, HER2, CK5/6, and EGFR with a further four studies providing data on ER, PR, HER2, and CK5/6, but not EGFR. Based on these data, there were 10,159 subjects that could be classified into one of the five major breast subtypes. There were 3,181 deaths in 85,799 person-years of follow-up, with 1,975 deaths from breast cancer. The multivariate, period-specific hazard ratios for age (in four categories), tumour grade, tumour size, node status, and the IHC markers are given in Table 2. These data show that the hazard ratios for all variables except age at diagnosis attenuate over time, and that for ER, PR, HER2, CK5/6, EGFR, and grade the effect changes direction with time. The time-dependent changes were most pronounced for ER and PR status. There was little difference in the hazard ratios for all-cause mortality and breast cancer-specific mortality, except for in the youngest and oldest age groups (Figures S1 and S2). Breast cancer-specific hazard ratios tended to be higher for women diagnosed under the age of 40 y (reference age at diagnosis 50–59 y). In contrast, for age at diagnosis ≥60 y, all-cause mortality hazard ratios were greater, as might be expected because of the impact of mortality from other causes.

**Table 2:** Multivariate period-specific all-cause mortality hazard ratios (95% CI).

Variable	Time after Diagnosis				
	0–2 y	2–4 y	4–6 y	6–10 y	10–15 y
**Age at diagnosis (y)**					
<40	0.69 (0.49–0.98)	1.09 (0.87–1.37)	1.14 (0.84–1.55)	0.83 (0.62–1.12)	0.68 (0.44–1.05)
40–49	0.63 (0.48–0.84)	0.77 (0.64–0.93)	0.83 (0.65–1.06)	0.66 (0.53–0.82)	0.51 (0.38–0.68)
50–59	1.00 (ref)	1.00 (ref)	1.00 (ref)	1.00 (ref)	1.00 (ref)
≥60	1.74 (1.36–2.22)	1.26 (1.04–1.52)	1.64 (1.31–2.06)	1.79 (1.49–2.14)	2.05 (1.63–2.58)
**Grade** a	1.51 (1.24–1.84)	1.81 (1.59–2.08)	1.37 (1.18–1.60)	1.14 (1.01–1.29)	0.97 (0.83–1.13)
**Node positive**	2.64 (2.12–3.27)	2.42 (2.09–2.82)	1.86 (1.55–2.23)	1.56 (1.35–1.82)	1.40 (1.15–1.70)
**Tumour size** a	1.67 (1.42–1.97)	1.47 (1.31–1.66)	1.43 (1.23–1.66)	1.37 (1.20–1.56)	1.30 (1.09–1.55)
**ER positive**	0.55 (0.42–0.71)	0.76 (0.63–0.91)	1.31 (1.02–1.68)	1.63 (1.29–2.07)	1.24 (0.91–1.69)
**PR positive**	0.36 (0.27–0.47)	0.62 (0.52–0.74)	0.74 (0.6–0.91)	1.04 (0.87–1.23)	1.16 (0.92–1.46)
**HER2 positive**	1.21 (0.95–1.52)	1.50 (1.27–1.78)	1.55 (1.23–1.96)	1.35 (1.07–1.69)	0.96 (0.67–1.37)
**Basal marker positive**	1.33 (1.06–1.68)	1.21 (1.01–1.44)	1.38 (1.08–1.78)	1.06 (0.83–1.35)	0.83 (0.59–1.17)

All analyses are stratified by study.

a Grade and tumour size are ordinal variables treated as continuous, giving hazard ratios per unit increase in score.

There were 7,882 luminal tumours (78% of total). Of these, 7,243 (92%) were luminal 1 and 639 (8%) were luminal 2. There were 632 tumours of the nonluminal HER2-positive subtype (6% of total), and 1,645 TNP tumours (16% of total). Of the TNP tumours, 962 were CBP (58%) and 683 basal-negative tumours (42%). The number of tumours by the five major subtypes for each study are shown in Table 3. In addition to the five main subtypes, we subdivided the luminal 1 tumours according to expression of basal markers, with 562 (8%) being basal marker positive and 6,119 (92%) being basal marker negative (Table S2 shows the luminal 1 subgroups by study). Table 4 shows the characteristics of the five major breast cancer subtypes by age at diagnosis, tumour grade, tumour size, and node status.

**Table 3:** Number of tumours by subtype and study.

Study	Luminal 1		Luminal 2		Nonluminal HER2+		CBP		5NP		Total
	*n*	Percent	*n*	Percent	*n*	Percent	*n*	Percent	*n*	Percent	
**ABCS**	497	67	64	9	51	7	60	8	68	9	740
**BCCA**	2,378	71	206	6	238	7	317	9	209	6	3,348
**HEBCS**	169	72	25	11	8	3	21	9	13	6	236
**JGH**	160	77	18	9	5	2	21	10	3	1	207
**MCBCS**	219	86	24	9	4	2	8	3	1	<1	256
**MCCS**	276	72	22	6	30	8	37	10	17	4	382
**NOBCS**	1,051	71	44	3	71	5	196	13	108	7	1,470
**PBCS**	694	69	35	3	67	7	137	14	75	7	1,008
**SBCS**	206	77	16	6	10	4	14	5	21	8	267
**SEARCH**	1,247	76	121	7	71	4	112	7	83	5	1,634
**UBC**	154	42	53	15	62	17	15	4	81	22	365
**VGH**	192	78	11	4	15	6	24	10	4	2	246
**Total**	7,243	71	639	6	632	6	962	9	683	7	10,159

ABCS, Amsterdam Breast Cancer Study; BCCA, British Columbia Cancer Agency; HEBCS, Helsinki Breast Cancer Study; JGH, Jewish General Hospital; MCBCS, Mayo Clinic Breast Cancer Study; MCCS, Melbourne Collaborative Cohort Study; NOBCS, Nottingham Breast Cancer Case Series; PBCS, Polish Breast Cancer Study; SBCS, Sheffield Breast Cancer Study; SEARCH, Study of Epidemiology and Risk factors in Cancer Heredity; UBCBCT, University of British Columbia Breast Cancer Trials; VGH, Vancouver General Hospital.

**Table 4:** Characteristics of breast cancer subtypes by age at diagnosis, tumour grade, tumour size, and node status.

Breast Cancer Subtype Characteristics	Luminal 1		Luminal 2		HER2-enriched		CBP		5NP		Total *n*	Percent
	*n*	Percent	*n*	Percent	*n*	Percent	*n*	Percent	*n*	Percent		
**Vital status at censoring**												
Alive	5,242	72	369	58	333	53	590	61	444	65	6,978	69
Dead	2,001	28	270	42	299	47	372	39	239	35	3,181	31
**Age group (y)**												
<40	457	6	74	12	80	13	165	17	90	13	866	9
40–49	1,960	27	215	34	190	30	286	30	237	35	2,888	28
50–59	3,142	43	233	36	268	42	377	39	238	35	4,258	42
≥60	1,684	23	117	18	94	15	134	14	118	17	2,147	21
**Tumour grade**												
1	1,493	21	41	6	20	2	15	3	40	6	1,609	16
2	3,645	50	239	37	146	23	129	13	174	25	4,333	42
3	2,105	29	359	56	466	73	818	85	469	69	4,217	42
**Node status**												
Negative	4,229	58	278	44	267	42	577	60	367	54	5,718	56
Positive	3,014	42	361	56	365	58	385	40	316	46	4,441	44
**Tumour size**												
<2 cm	4,441	61	300	47	272	43	442	46	296	43	5,751	56
2–4.9 cm	2,580	36	306	48	318	50	468	49	336	49	4,008	39
≥5 cm	222	3	33	5	42	7	52	5	51	7	402	4

**Figure 2:**
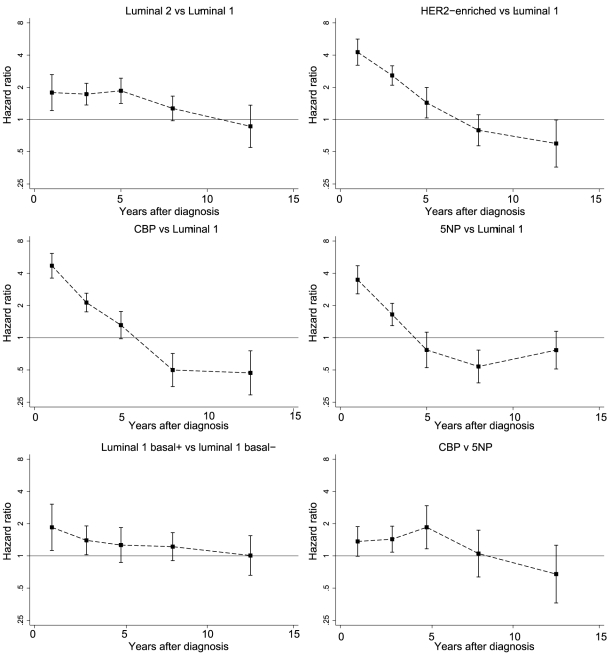
Period-specific hazard ratios (all-cause mortality) for major breast cancer subtypes. All hazard ratios are stratified by study and adjusted for tumour grade, tumour size, and node status.

The hazard ratios over time for the five subtypes of breast cancer, stratified by study and adjusted for grade, tumour size, and node status, are shown in Figure 2. There was little evidence for heterogeneity of effects by study for these hazard ratios except for the 5NP tumours (Table S3). Figure 2 shows that, compared to the luminal 1 tumours, luminal 2 tumours are associated with a slightly poorer prognosis in the first few years after diagnosis, but that the difference reduces with time, and by 8 y after diagnosis there is no difference between the two. In contrast the mortality for women with the HER2-enriched and both types of TNP tumours (CBP and 5NP) is substantially greater than that for women with the luminal 1 tumours immediately after diagnosis, but the difference declines rapidly and reverses at 5–10 y after diagnosis. These patterns reflect the time-dependent changes in mortality rates in the different subgroups (Figure S3). Within the TNP subgroup, the women with CBP tumours have a slightly poorer prognosis than women with the 5NP tumours. This difference declines slightly over time and by 8 y after diagnosis, no difference is observed. A similar pattern is seen for the luminal 1, basal-positive tumours when compared to the luminal 1, basal-negative tumours. We repeated the analyses using breast cancer-specific mortality as the end point (Figure S4). The hazard ratio estimates tended to be greater (for hazard ratios greater than unity) than the all-cause mortality hazard ratios, but the confidence intervals were somewhat wider.

**Figure 3:**
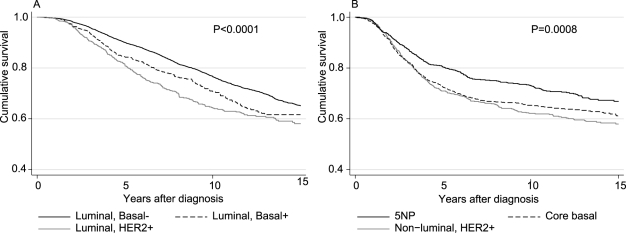
(A and B) Kaplan-Meier cumulative survival (all-cause mortality) in luminal and nonluminal tumours by subtype. All curves are adjusted for age at diagnosis, tumour grade, tumour size, node status, and study.

The Kaplan-Meier cumulative survival for the three luminal subtypes adjusted for study, grade, tumour size, and node status is shown in Figure 3A. This result shows that the cumulative survival for the luminal 1 subtypes declines almost linearly over time, which is compatible with a constant mortality rate. In contrast, the mortality rate in women with the luminal 2 tumours tends to flatten out over time as the high mortality in the first few years after diagnosis declines. It also clearly shows the poorer prognosis for the luminal 1 tumours that are basal marker positive. The survival curves associated with nonluminal HER2-positive, CBP, and 5NP tumours all show a similar pattern to that of the luminal 2 tumours (Figure 3B). There were significant differences in prognosis between all pairs of subtypes apart from the nonluminal HER2-positive tumours compared with the CBP tumours (Table S4). Of particular note is the difference between the CBP and 5NP tumours (*p* = 0.0008). The luminal, HER2-positive tumours and the nonluminal, HER2-positive tumours are two distinct subgroups, with the nonluminal tumours having a poorer prognosis (*p*<0.0001), and the CBP tumours having a poorer prognosis than the luminal, basal-positive tumours (*p*<0.0001). These differences did not depend on whether or not the patient had been treated with either adjuvant hormone therapy or adjuvant chemotherapy (Figure S5). In contrast, the basal markers seem to have no prognostic significance within the HER2 positive subtypes of disease (*p* = 0.85).

The luminal, HER2-positive tumours and the nonluminal, HER2-positive tumours represent two distinct subgroups, as do the ER-positive/negative tumours that are basal positive. In both cases the ER-negative tumours have a poorer prognosis in the first few years after diagnosis, but after 5 to 10 y it is the ER-positive tumours that have the poorer outcome (Figure S6). In contrast, the basal markers seem to have no prognostic significance within the HER2-positive subtypes of disease (unpublished data).

Data on the association between the major subtypes and prognosis have previously been published for three of the studies included in this analysis—BCCA, JGH, and VGH—and it is possible that the effect estimates that we report here are subject to publication bias. We therefore repeated all the analyses after excluding the data for these three studies but there was little difference in the results (see Figure S7).

## Discussion

We evaluated the prognostic significance of five previously described major subtypes of breast cancer that were classified using five IHC markers. To our knowledge, this study represents one of the largest datasets analysed for prognosis research in breast cancer using IHC markers. Our data confirm the observations of others that the pattern of survival in ER-positive tumours is qualitatively different to that in ER-negative tumours. In ER-positive tumours, the mortality rate is approximately constant over time since diagnosis, whereas the mortality rate associated with ER-negative disease is initially high and then progressively declines over time. However, the pattern of mortality rates associated with the HER2-positive subgroup of ER-positive tumours (luminal 2) is similar to those of the nonluminal subtypes (Figure 3A).

Berry et al. suggest [14] that the pattern of mortality after diagnosis associated with ER-positive tumours is mainly an effect of treatment with adjuvant hormone therapy and that the pattern of mortality in women not treated with adjuvant hormone therapy is similar to that in women with ER-negative disease. The pattern of mortality in women with luminal 1 tumours and treated with adjuvant hormone therapy was similar to those who did not receive hormone therapy (Figure S3). This result implies that the time-dependent effects we observed are not simply the result of adjuvant hormone therapy in a subset of the women with ER-positive tumours. Few of the participants with HER2-positive tumours in this study would have been treated with trastuzumab and so the prognosis in women with these tumours would not reflect the benefit of targeted therapy. Instead we propose that the survival patterns reflect the underlying molecular heterogeneity of breast cancer. We have hypothesized that this heterogeneous biology reflects the fact that breast cancers can initiate in different cell types, either breast epithelial stem cells or their progeny (transit amplifying cells or committed differentiated cells) [30]. Furthermore the recognition of the subtype-specific differences in short-term and long-term prognosis will inevitably lead to tailored follow-up programmes after completion of primary therapy.

Our data confirm the view that the TNP is not a good proxy for the CBP because the CBP and 5NP tumours are biologically distinct and show different behaviours. The CBP tumours are clearly associated with a poorer prognosis than the 5NP tumours. Currently, chemotherapy remains the only systemic treatment option available for patients with triple negative (CBP and 5NP) tumours. A number of small studies have shown that basal-like cancers defined through gene-expression profiling or immunophenotyping are responsive to chemotherapy regimes [31]–[33]. In addition, the expression of core basal markers such as EGFR, may lead to the application of targeted therapies, with EGFR inhibitors currently under investigation for use in basal-like breast cancers. We have also shown that the expression of basal markers in ER-positive tumours is associated with a poorer prognosis, suggesting that the luminal 1 tumours represent two distinct subtypes, both of which differ in behaviour from the luminal 2 tumours. Overall the prognostic model based on the six subtypes defined by five IHC markers fits significantly better than a model based on three subtypes—ER-positive or PR-positive and HER2-negative, HER2-positive, and triple-negative tumours—defined by the three markers currently in standard clinical practice (likelihood ratio chisq = 54.4, 3 degrees of freedom [df], *p*<0.0001).

One remaining question is whether the 5NP tumours represent a distinct subtype or are just other subtypes that have been misclassified because of assay failure. However, given the pattern of mortality rates over time since diagnosis (Figure S3), it seems unlikely that many of the 5NP tumours are misclassified luminal tumours. If the 5NP tumours were misclassified nonluminal HER2-positive or CBP tumours, we would expect the survival associated with them to be intermediate, whereas the 5NP tumours have a better prognosis than both the other nonluminal subtypes. Furthermore, the prognosis associated with the 5NP is different from each of the other five subtypes and is also different from all the other subtypes combined. Thus it seems likely that the majority of 5NP tumours represent a true distinct subtype, with a small, but unknown, proportion representing misclassification of the other subtypes, Until a marker to positively identify the genuine 5NP subtype has been identified, it will not be possible to separate these two sets of tumours.

Our study has several limitations. IHC was carried out in different laboratories using different methods for both staining and scoring and, as a result, some misclassification of tumour subtypes is inevitable. However, it is likely that such error is random with respect to patient outcome. For the analyses of breast cancer-specific mortality, cause of death was obtained from the underlying cause of death as reported on death certificates and may thus be associated with some error. However, any error in ascertaining cause of death is likely to be random with respect to tumour characteristics. Thus, measurement error of either breast cancer subtype, as a result of interlaboratory variability or outcome, is, if anything, likely to result in an underestimate of any true differences between subtypes. The fact that we have found clear differences in subtypes classified by IHC analyses that were carried out in different laboratories, and would therefore be subject to interlaboratory assay result variability, suggests that the markers are robust to interlaboratory variation in their application and therefore suitable for use in routine clinical practice.

There is also some nonrandom error as the luminal 1 tumours that express proliferation markers are likely to behave more like luminal 2 tumours [6]. As the luminal 1 tumours were used as the reference category, this misclassification is likely to lead to an underestimation in the true difference between luminal 1 and the other subtypes. Similarly, some of the 768 5NP tumours will be misclassified CBP tumours because data on EGFR were missing. Assuming these data were missing at random, approximately 25 of the 5NP tumours may represent misclassified CBP tumours. However, when the definition of 5NP tumours was restricted to those that were negative for both CK5/6 and EGFR, there was little difference in the hazard ratio estimates (unpublished data. Finally, the effects may also be underestimated because of the nonrandom use of adjuvant chemotherapy. The more aggressive subtypes are more likely to have been treated with chemotherapy, which would result in a reduction in the difference between these groups and the better prognosis subtypes.

Data from 12 different studies were used in this analysis. These studies represent different ethnic groups from different regions of the world as well as differences in case ascertainment. Furthermore there were differences in the way that pathology samples were handled, stained, and scored, and the degree of misclassification will vary from study to study. This heterogeneity in study design may weaken the observed associations, and limit the specificity of the conclusions drawn. Nevertheless, the clear differences between the subtypes of breast cancer that we identified, despite the presence of heterogeneity, make the results robust and broaden their generalisability.

In conclusion, we have confirmed that six breast cancer subtypes can be robustly classified using five IHC markers. These subtypes behave differently with specific patterns of mortality over time since diagnosis. These characteristics are independent of other clinico-pathological markers of prognosis and independent of systemic therapy received. The classification based on these markers is robust to multiple sources of heterogeneity between studies suggesting that they are suitable for use in routine clinical practice. The incorporation of these markers into prognostic tools such as Adjuvant!Online and the Nottingham Prognostic Index currently used in clinical practice or tools such as PREDICT [34], which was recently developed to enable the incorporation of novel prognostic biomarkers, may be warranted. It is plausible that these markers are predictive and that different subtypes respond differently to specific treatments, and the evaluation of subtype-specific responses in the context of clinical trials of specific treatments is urgently required. Given that these subtypes can easily be defined using robust IHC markers in archival material, this type of analysis should be possible with existing clinical trial data.

## Supporting Information

Figure S1 Comparison of multivariate, period-specific hazard ratios for age group, tumour grade, and node status based on all-cause and breast-specific mortality. Left-hand panel are results for all-cause mortality and right-hand panels results for breast-specific mortality. Tumour size was treated as an ordinal variable in the Cox regression models and so the hazard ratios represent the hazard ratio for a unit change in the variable.
(1.29 MB EPS)

Figure S2 Comparison of multivariate, period-specific hazard ratios for tumour size, ER, PR, HER2, and basal marker status based on all-cause and breast-specific mortality. Left-hand panel are results for all cause mortality and right-hand panels results for breast specific mortality. Tumour size was treated as ordinal variables in the Cox regression models and so the hazard ratios represent the hazard ratio for a unit change in the variable.
(1.26 MB EPS)

Figure S3 Breast cancer-specific mortality by subtype and time since diagnosis.
(0.65 MB EPS)

Figure S4 Period-specific hazard ratios (breast-specific mortality) for major breast cancer subtypes. All hazard ratios are stratified by study and adjusted for tumour grade, tumour size, and node status.
(1.01 MB EPS)

Figure S5 Kaplan-Meier cumulative survival in luminal and nonluminal tumours by subtype and by treatment with adjuvant hormone therapy and adjuvant chemotherapy. All curves are adjusted for age at diagnosis, tumour grade, tumour size, node status, and study.
(2.18 MB EPS)

Figure S6 Period-specific hazard ratios for ER-negative versus ER-positive disease stratified by HER2 status and basal marker status. All hazard ratios are adjusted for age at diagnosis, tumour grade, tumour size, and node status and stratified by study.
(0.81 MB EPS)

Figure S7 Comparison of period- and subtype-specific hazard ratios (all-cause mortality) for all data and for subset of data after excluding published studies. Left-hand panels show results based on all data (as shown in Figure 1) and right-hand panels show equivalent hazard ratios after exclusion of data from BCCA, JGH, and VGH.
(1.24 MB EPS)

Table S1 Methods used for IHC analysis by study.
(0.10 MB DOC)

Table S2 Classification of luminal 1 tumours by basal marker expression.
(0.04 MB DOC)

Table S3 *p*-Values for test for heterogeneity of period-specific hazard ratio estimates (compared to luminal 1 tumours) by study.
(0.03 MB DOC)

Table S4 Likelihood ratio test statistic (2 degrees of freedom) and p-value for comparison of 15-y all-cause mortality between each subtype pair.
(0.04 MB DOC)

## Abbreviations

5NP: five negative phenotype
BCAC: international breast cancer association consortium
CBP: core basal subgroup
CK: cytokeratin
EGFR: epidermal growth factor receptor
ER: estrogen receptor
HER2: human epidermal growth factor receptor-2
IHC: immunohistochemical
PR: progesterone receptor
TNP: triple negative phenotype
