# Supplementary material for: Subtyping of Breast Cancer by Immunohistochemistry to Investigate a Relationship between Subtype and Short and Long Term Survival: A Collaborative Analysis of Data for 10,159 Cases from 12 Studies
Source: PLoS Med. 2010 May 25;7(5):e1000279. doi: 10.1371/journal.pmed.1000279 (PMC2876119; doi:10.1371/journal.pmed.1000279)
Supplement: Table S1 — Methods used for IHC analysis by study. (0.10 MB DOC) [file pmed.1000279.s008.doc]

Table S1: Methods used for immunohistochemical analysis by study

| **Study** | **Antibody** | **Supplier** | **Clone** | **Dilution** | **Source** | **Definition of positivity** |
| --- | --- | --- | --- | --- | --- | --- |
| ABCS | CK5-6 | Dako | D5/16 B4 | 1:100 | T | >1% cells staining |
| ABCS | ER | Neomarkers, Labvision | 1D5 and 6F11 | 1:50 | T | >10% nuclei staining |
| ABCS | HER2 | NeoMarkers, LabVision | 3B5 and 23 | 1:400 | T | Intensity 3+ |
| ABCS | PR | ImmunoLogic | PR-1 | 1:500 | T | >10% nuclei staining |
| BCCA | CK5-6 | Zymed | D5/16B4 |  | T | Any cytoplasmic or membrane staining |
| BCCA | EGFR | Dako | pharmDx kit |  | T | Any cytoplasmic or membrane staining |
| BCCA | ER | LabVision | SP1 |  | T | >1% nuclei staining |
| BCCA | HER2 | LabVision | SP3 |  | T | Intensity 3 or intensity 2 and FISH+ |
| BCCA | PR | Ventana | 1E2 |  | T | >1% nuclei staining |
| HEBCS | CK5-6 | Dako | M7237 | 1:25 | T | >10% cells staining |
| HEBCS | EGFR | Zymed | 31G7 | 1:500 | T | >10% cells staining |
| HEBCS | ER | Novocastra |  | 1:50 | R | >10% nuclei staining |
| HEBCS | HER2 | Novocastra | NCL-CB11 | 1:2000 | T | Intensity 3 or intensity 2 and CISH+ |
| HEBCS | PR | Dako | PgR 636 | 1:250 | R | >10% nuclei staining |
| JGH | CK5-6 | Dako | D5/16B4 | 1:25 | S | Any cytoplasmic or membrane staining |
| JGH | EGFR | Dako | pharmDx kit | Pre-diluted | T | Any cytoplasmic or membrane staining |
| JGH | ER | Ventana | 6F11 | Pre-diluted | S | >10% nuclei staining |
| JGH | HER2 | Signet Pathology Systems | CB11 | 1:100 | S | Intensity 2 or 3 |
| JGH | PR | Ventana | 1A6 | Pre-diluted | S | >10% nuclei staining |
| MCBCS | CK5-6 | Zymed | D516B4 | 1:200 | S | >10% cells staining |
| MCBCS | ER | Novocastra | 6F11/2 | 1:20 | S | >1% nuclei staining |
| MCBCS | HER2 | Dako | HercepTest™ | Pre-diluted | S | Intensity 3 in >30% cells |
| MCBCS | PR | Dako | PgR 636 | 1:100 | S | >1% nuclei staining |
| MCCS | CK5-6 | Dako M7237 | D5/16 B4 | 1:250 | S | Any cytoplasmic or nuclear staining |
| MCCS | EGFR | Zymed 28-005 | 31G7 | 1:1000 | S | Any cytoplasmic or nuclear staining |
| MCCS | ER | Neomarkers RM9101 | SP1 clone | 1:250 | S | >5% nuclei staining |
| MCCS | HER2 | Dako A0485 | cerB2 | 1:1000 | S | Intensity 2 or 3 |
| MCCS | PR | Dako M3569 | PgR 636 | 1:1000 | S | I>5% nuclei staining |
| NOBCS | CK5-6 | Boehringer Biochemica | D5/16134 | 1:100 | T | >10% cells staining |
| NOBCS | EGFR | Novocastra | EGFR.113 | 1:10 | T | >10% cells staining |
| NOBCS | ER | Novocastra | 6F11/2 | 1:30 | T | >10% nuclei staining |
| NOBCS | HER2 | DAKO | cerbB-2 | 1:250 | T | >10% cells staining |
| NOBCS | PR | Dako | PgR 636 | 1:50 | T | >10% nuclei staining |
| PBCS | CK5 | Novocastra | D5/16 B4 | 1:100 | T | Product of intensity (0-3) and percentage (0-100) >10 |
| PBCS | EGFR | Zymed | 31G7 | 1:500 | T | Product of intensity (0-3) and percentage (0-100) >10 |
| PBCS | ER | Novocastra | 6F11/2 (1D5 for AQUA) | 1:200 | T and R | Product of intensity (0-3) and percentage (0-100) >10 |
| PBCS | HER2 | Dako |  | 1:2000 | T | Intensity 3 in >20% cells |
| PBCS | PR | Dako | PgR 636 | 1:1000 | T and R | Product of intensity (0-3) and percentage (0-100) >10 |
| SBCS | CK5-6 | Vector | XM26 | 1:50 | T | >10% cells staining |
| SBCS | ER | Vector | 6F11/2 | 1:50 | T and R | Product of intensity (0-3) and percentage (0-100) >=50 |
| SBCS | HER2 | Dako | HercepTest kit K5204 | Pre-diluted | T | Intensity 2 or 3 |
| SBCS | PR | Vector | 1A6 | 1:40 | T | Sum of intensity (0-3) and percentage (0-5) >=3 |
| SEARCH | CK14 | Novocastra | LL002 | 1:20 | T | >10% cells staining |
| SEARCH | CK5-6 | Dako | D5/16 B4 | 1:50 | T | >10% cells staining |
| SEARCH | EGFR | Zymed | 31G7 | 1:25 | T | >10% cells staining |
| SEARCH | ER | Novocastra | 6F11/2 | 1:70 | T and R | Sum of intensity (0-3) and percentage (0-5) >=3 |
| SEARCH | HER2 | Dako | Herceptest kit K5207 | Pre-diluted | T and R | Intensity 2 or 3 |
| SEARCH | PR | Dako | PgR 636 | 1:50 | T and R | Sum of intensity (0-3) and percentage (0-5) >=3 |
| UBCBCS | CK5-6 | Dako | D5/16 B4 | 1:100 | T | >10% cells staining |
| UBCBCS | EGFR | Zymed | 31G7 | 1:25 | T | >10% cells staining |
| UBCBCS | ER | Novocastra | 6F11/2 | 1:30 | T and R | >10% cells staining |
| UBCBCS | HER2 | Novocastra | CBE-356 | 1:40 | T and R | Intensity 2 or 3 |
| UBCBCS | PR | Dako | PgR 636 | 1:50 | T and R | >10% cells staining |
| VGH | CK5-6 | Dako | D5/16B4 | 1:25 | T | Any cytoplasmic staining |
| VGH | EGFR | Dako | pharmDx kit | Pre-diluted | T | Any cytoplasmic or membrane staining |
| VGH | ER | Ventana | 6F11 | Pre-diluted | T | >10% nuclei staining |
| VGH | HER2 | Signet Pathology Systems | CB11 | 1:100 | T | Intensity 2 or 3 |
| VGH | PR | Ventana | 1A6 | Pre-diluted | T | >10% nuclei staining |
