# Supplementary material for: Subtyping of Breast Cancer by Immunohistochemistry to Investigate a Relationship between Subtype and Short and Long Term Survival: A Collaborative Analysis of Data for 10,159 Cases from 12 Studies
Source: PLoS Med. 2010 May 25;7(5):e1000279. doi: 10.1371/journal.pmed.1000279 (PMC2876119; doi:10.1371/journal.pmed.1000279)
Supplement: Table S2 — Classification of luminal 1 tumours by basal marker expression. (0.04 MB DOC) [file pmed.1000279.s009.doc]

Table S2: Classification of luminal 1 tumours by basal marker expression

| **Study** | **Basal -** | **%** | **Basal+** | **%** | **Total** |
| --- | --- | --- | --- | --- | --- |
| ABCS | 417 | 97 | 11 | 3 | 428 |
| GPEC | 2,125 | 95 | 107 | 5 | 2232 |
| HEBCS | 134 | 82 | 30 | 18 | 164 |
| JGH | 103 | 67 | 50 | 33 | 153 |
| MCCS | 267 | 97 | 9 | 3 | 276 |
| NOBCS | 886 | 84 | 165 | 16 | 1051 |
| PBCS | 633 | 92 | 54 | 8 | 687 |
| SBCS | 180 | 93 | 14 | 7 | 194 |
| SEARCH | 1,058 | 91 | 102 | 9 | 1160 |
| UBC | 152 | 99 | 1 | 1 | 153 |
| VGH | 164 | 90 | 19 | 10 | 183 |
| Total | 6,119 | 92 | 562 | 8 | 6,681 |
