# Supplementary material for: Subtyping of Breast Cancer by Immunohistochemistry to Investigate a Relationship between Subtype and Short and Long Term Survival: A Collaborative Analysis of Data for 10,159 Cases from 12 Studies
Source: PLoS Med. 2010 May 25;7(5):e1000279. doi: 10.1371/journal.pmed.1000279 (PMC2876119; doi:10.1371/journal.pmed.1000279)
Supplement: Table S3 — p-Values for test for heterogeneity of period-specific hazard ratio estimates (compared to luminal 1 tumours) by study. (0.03 MB DOC) [file pmed.1000279.s010.doc]

Table S1: P-values for test for heterogeneity of period specific hazard ratio estimates (compared to luminal 1 tumours) by study

| Period | Luminal 2 | Non-luminal HER2 positive | CBP | 5NP |
| --- | --- | --- | --- | --- |
| 0-2 years | 0.054 | 0.68 | 0.37 | 0.28 |
| 2-4 years | 0.57 | 0.82 | 0.22 | 0.017 |
| 4-6 years | 0.89 | 0.16 | 0.99 | 0.085 |
| 6-10 years | 0.43 | 0.018 | 0.50 | 0.022 |
| 10-15 years | 0.78 | 0.44 | 0.99 | 0.86 |
