# Supplementary material for: Subtyping of Breast Cancer by Immunohistochemistry to Investigate a Relationship between Subtype and Short and Long Term Survival: A Collaborative Analysis of Data for 10,159 Cases from 12 Studies
Source: PLoS Med. 2010 May 25;7(5):e1000279. doi: 10.1371/journal.pmed.1000279 (PMC2876119; doi:10.1371/journal.pmed.1000279)
Supplement: Table S4 — Likelihood ratio test statistic (2 degrees of freedom) and p-value for comparison of 15-y all-cause mortality between each subtype pair. (0.04 MB DOC) [file pmed.1000279.s011.doc]

Table S4: Likelihood ratio test statistic (2 d.f.) and P-value for comparison of 15-year all cause mortality between each subtype pair

|  | Luminal 1, basal positive | Luminal 2 | Non-luminal, HER2 positive | CBP | 5NP |
| --- | --- | --- | --- | --- | --- |
| Luminal 1, basal negative | 14.1 | 48.2 | 162.9 | 195.9 | 86.7 |
|  | P=0.0009 | P<0.0001 | P<0.0001 | P<0.0001 | P<0.0001 |
| Luminal 1, basal positive |  | 7.16 | 24.7 | 41.9 | 20.9 |
|  |  | P=0.028 | P<0.0001 | P<0.0001 | P<0.0001 |
| Luminal 2 |  |  | 35.5 | 44.9 | 29.9 |
|  |  |  | P<0.0001 | P<0.0001 | P<0.0001 |
| Non-luminal, HER2 positive |  |  |  | 3.91 | 15.1 |
|  |  |  |  | P=0.014 | P=0.0001 |
| CBP |  |  |  |  | 14.2 |
|  |  |  |  |  | P = 0.0008 |

All comparisons stratified by study and adjusted for age at diagnosis, tumour grade, tumour size and node status
